# Risk Assessment of the Schmutzdecke of Biosand Filters: Identification of an Opportunistic Pathogen in Schmutzdecke Developed by an Unsafe Water Source

**DOI:** 10.3390/ijerph110202033

**Published:** 2014-02-14

**Authors:** Hyun Gyu Hwang, Min Seo Kim, Soo Min Shin, Cher Won Hwang

**Affiliations:** 1 School of Life Science, Handong Global University, Gyeongsangbuk-do, Pohang 791-708, Korea; E-Mails: gusrbcpt90@naver.com (H.G.H.); minseolike@naver.com (M.S.K.); soomin.shin@hotmail.com (S.M.S.); 2 Global Leadership School, Handong Global University, Gyeongsangbuk-do, Pohang 791-708, Korea

**Keywords:** opportunistic pathogen, nearest phylogenetic neighbor, biosand filter, schmutzdecke, 16S rRNA gene sequencing

## Abstract

The biosand filter (BSF) is widely applied in developing counties as an appropriate technology-based product for supplying “safe” water. Biosand filters exhibit relatively high purifying efficiency because of the schmutzdecke (biofilm) embedded in them. However, schmutzdecke should be cleaned or discarded on a regular basis to maintain the purifying efficiency of the BSF. Due to its role in BSFs, the purifying function of schmutzdecke, rather than its potential risk when not properly discarded, has so far been the primary focus of research. This study aims to provide a risk assessment of schmutzdecke in an attempt to draw attention to a wholly new angle of schmutzdecke usage. We conducted 16S rRNA gene sequencing and phylogenetic analysis to identify opportunistic pathogens in schmutzdecke developed using water from the Hyung-San River. The results reveal that the schmutzdecke derived from this water source contains diverse and relatively high portions of opportunistic pathogen strains; 55% of all isolates collected from schmutzdecke were identified as opportunistic pathogens. Moreover, the diversity of microorganisms is increased in the schmutzdecke compared to its water source in terms of diversity of genus, phylum and opportunistic pathogen strain. As a whole, our study indicates a potential risk associated with schmutzdecke and the necessity of a solid guideline for the after-treatment of discarded schmutzdecke.

## 1. Introduction

Many developing countries are facing water problems, both in terms of lacking sufficient supplies of water and producing “clean” water. It is reported that more than 1.1 billion people are suffering from a lack of safe water [1]. To solve this problem, developing countries need to build a solid base for a water treatment system. However, such systems are very expensive, and thus, an alternative solution using “appropriate technology” is attracting attention [2].

Appropriate technology, also known as intermediate technology, is a term describing a technology that is designed to provide minimal technical challenges to people living in circumstances where high technology is difficult to apply. Differing from high technology, appropriate technology uses simple and low-cost materials that are easily obtainable in the locale and does not require specialized techniques [3]. Due to these characteristics, appropriate technology is increasingly frequently applied in developing countries.

The biosand filter (BSF) is one of the most widely applied appropriate technology-based products. The 2012 Annual Report of the Centre for Affordable Water and Sanitation Technology (CAWST) presents statistical data indicating that 5,981,000 people are impacted by the water sanitation project encompassing biosand filters (BSF) [4]. Furthermore, according to the CAWST database, over 200,000 BSFs have been installed globally so far, 12,346 institutions are participating in supplying BSFs, and 37 countries are taking advantage of BSF systems [5].

**Figure 1:**
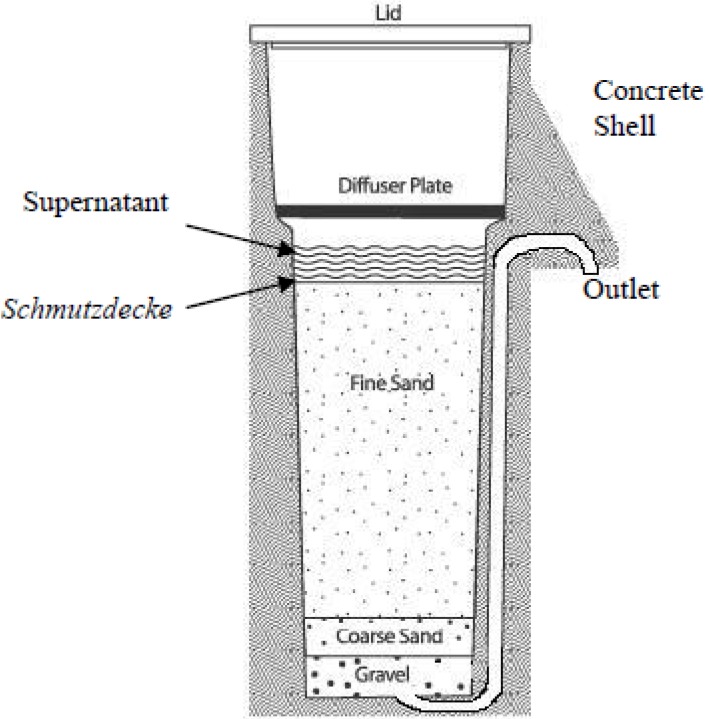
Biosand filter (source: [11]).

Even though the biosand filter is composed of easily obtainable and simple materials, such as sand, it exhibits relatively high biological purification efficiency, removing 93–99% of fecal coliform bacteria [6] and 99.9% of protozoa [7]. Underlying the relatively high biological purifying effect of the BSF is its schmutzdecke (biofilm). BSF provides an appropriate condition for the growth of microorganisms, encouraging them to form a biofilm called schmutzdecke (Figure 1). The microorganisms composing the schmutzdecke, in turn, prey on other harmful microorganisms contained in the contaminated input water, converting them to harmless inorganic matter [8]. Moreover, various microorganisms from the input water source attach to the sand surface and accumulate to become part of schmutzdecke [9]. The participation of water source-originated microorganisms in schmutzdecke further increases the biological purification efficacy.

Because schmutzdecke clogs and prevents water flow through the filter as it develops, it should be cleaned or discarded on a regular basis to maintain the BSF [10]. As a general rule, people using BSFs dump or pour waste of the schmutzdecke to nearby water sources, such as ditches, lakes, or rivers, for their convenience and without much awareness. This practice has continued because there have been no solid guidelines for the after-treatment of schmutzdecke deposits. No one has cast any doubts on the traditional method of discarding schmutzdecke and whether it possesses potential risk. Due to its role in the BSF, so far, the main focus of schmutzdecke research has been its purifying function rather than the potential risks it poses. Because it examines the potential negative side of schmutzdecke, which has thus far been neglected, this paper is expected to bring attention to an entirely new aspect of schmutzdecke. This paper aims to perform a risk assessment of schmutzdecke by evaluating the opportunistic pathogens and suggesting the necessity of guidelines for the after-treatment of schmutzdecke.

## 2. Experimental Section

### 2.1. Source of Samples

#### 2.1.1. Sample Collection from Hyung-San River

Samples were collected from two different sources: the Hyung-San River (latitude: 36.006826/longitude: 129.361632) in Pohang, South Korea, and the schmutzdecke of biosand filters. Water was collected at 30 cm below the surface of the Hyung-San River on 23 September 2013, and a basic water condition test was conducted within 2 hours. The results were as follows: pH 6.77, salinity 3.4 ppt, conductivity 6.28 ms/cm, resistance 0.002 MΩ-cm, SS 3075 mg/L, COD 3.4 mg/L, DO 8.23 mg/L. A 100 μL aliquot of the water sample was cultured in Plate Count Agar (BD DIFCO, 247940) medium in triplicate over a dilution range of 10^−1^–10^−7^. The 21 plates prepared (three sets of seven differently diluted plates) were incubated at 25 °C room temperature for 2 days, and 17 strains were isolated according to distinct colony morphologies.

#### 2.1.2. Sample Collection from the Schmutzdecke (Biofilm) of Biosand Filters

A minimized model of a biosand filter was constructed according to the manual offered by CAWST [10]. A container composed of polyethylene phthalate was used, and the specific parameters of the constructed minimized biosand filter were as follows: container height 30.0 cm, sand height 20.0 cm, container diameter 9.5 cm, diffuser hole diameter 0.3 cm, and sand grain size 0.7–1.0 mm. Twice a day, 300 mL of water was supplied to maintain a standing water zone of 5 cm above the sand surface with a pause period of 12 hours. The schmutzdecke (biofilm) embedded in BSF was developed for 4 weeks using the Hyung-San River as the water source. Schmutzdecke was mixed with PBS buffer and vortexed. Then, 100 μL of extracted sample was cultured in Plate Count Agar (BD DIFCO, 247940) medium in triplicate over a dilution range of 10^−1^–10^−7^. The 21 prepared plates were incubated at 25 °C room temperature for 2 days, and 20 strains were isolated according to distinct colony morphologies.

### 2.2. DNA Isolation

Each isolated sample was added to a 1.5 mL microtube, vortexed in DW and centrifuged for 1 min at 13,000 g. After removing the supernatant, 300 μL of cell resuspension solution (Solutions for Genetic Technology, Seoul, Republic of Korea) and 2 μL of lysozyme (100 mg/mL) were added, and the samples were incubated at 37 °C for 60 min. Again, the samples were centrifuged for 1 min at 13,000 g. Then, 300 μL of Cell Lysis Solution (Solutions for Genetic Technology) and 1.5 μL of RNase A (4 mg/mL) were added and mixed with the samples. After incubation at 37 °C for 30 min, the samples were cooled to room temperature, and 100 μL Protein Precipitation Solution (Solutions for Genetic Technology) was added. The samples were centrifuged for 5 min at 13,000 g, and the supernatants were transferred to new 1.5 mL microtubes containing 300 μL of 100% isopropanol. Then, the samples were washed twice with 500 μL of WB (80% Ethanol) with inverting. The supernatant liquid was removed with a micropipette, and the remaining pellet was dried at room temperature for 15 min. Subsequently, 100 μL of DNA Hydration solution (Solutions for Genetic Technology) was added, and the DNA was dissolved thoroughly through vortexing. The samples were incubated at 65 °C for 60 min, and the concentration of prepared DNA was confirmed through overnight electrophoresis.

### 2.3. PCR Amplification and Purification

Extracted DNA was amplified through PCR with a Veriti R ^TM^ 96-well Thermal Cycler (Applied Biosystems, Marsiling, Singapore), using the universal primer pair of 27F (5’-AGAGTTTGATCCTGGCTCAG-3’) and 1492R (5’-GGTTACCTTGTTACGACTT-3’). The DNA sample solution (3.0 μL) was mixed with 2.5 μL of 10x EF-Taq Buffer, 0.5 μL of 10 mM dNTP(T), 1.0 μL of primer (F10), 1.0 μL of primer (R10p), 0.3 μL of EF-Taq (2.5 U), and 16.7 μL of DW. The PCR protocol included a denaturation step at 95 °C for 15 min followed by 30 cycles of 3 constitutive steps: thermal cycling for 20 sec at 95 °C, 40 sec at 50°C, and 1 min and 30 sec at 72 °C. The final termination step was performed at 72 °C for 5 min. The amplified DNA was purified with an Ultra PCR Purification Kit (SolGent, Seoul, Republic of Korea) according to the manufacturer’s instructions.

### 2.4. 16S rRNA Gene Sequencing

The purified DNA (1.0 μL) from each strain was mixed with 4.0 μL of Terminator Ready Reaction Mix (Solutions for Genetic Technology), 1 μL of primer (5 pmol), and 4 μL of sterilized water, for a total reaction volume of 10 μL. Subsequently, the reaction tubes were subjected to the process of cycle sequencing, with 30 repeating thermal cycles at 96 °C for 10 sec, 50 °C for 5 seconds, and 60 °C for 4 min. Loading buffer (15 μL of Hi-Di Formamide) was added to 10 μL of each prepared reaction tube and then mixed and centrifuged during the final step. Each sample was heated for 4 min at 95 °C, immediately transferred to ice, and loaded on the ABI 3730XL DNA Analyzer (Applied Biosystems, Foster City, CA, USA) for sequencing.

### 2.5. Phylogenetic Analysis

Gene sequences for the 16S rRNA of the isolates were analyzed and identified for the nearest phylogenetic neighbor using the Basic Local Alignment Search (BLAST) tool from NCBI (National Center for Biotechnology Information, Bethesda, MD, USA) [12] and were aligned by using ClustalW in the MEGA software package (version 6.05). Neighbor-joining phylogenetic trees were constructed by the Maximum-Likelihood method in MEGA 6.05. Confidence values for nodes were measured using bootstrap resampling (1,000 replications) [13]. 

## 3. Results

### 3.1. Comparing the Diversity and Proportions of Microorganisms from Two Different Sources

Seventeen strains of bacteria were isolated from the Hyung-San River: *Novosphingobium, Catellibacterium, Aeromonas, Leclercia, Raoultella* from the phylum Proteobacteria and *Microbacterium* from the phylum Actinobacteria. Of these 17 strains, five isolates were identified as opportunistic pathogens (29%) according to the 16S rRNA-based phylogenic analysis. Twenty strains were isolated from the schmutzdecke of the BSF that was developed by supplying Hyung-San River for nutrients and organic matter: *Brevibacillus* from the phylum Firmicutes; *Cloacibacterium* from the phylum Bacteroidetes; *Streptomyces, Microbacterium,* and *Arthrobacter* from the phylum Actinobacteria; and *Novosphingobium, Sphingomonas, Bradyrhizobium, Klebsiella, Enterobacter, Aeromonas,* and *Pantoea* from the phylum Proteobacteria. Of the 20 strains, nine isolates were identified as opportunistic pathogen (55%) according to 16S rRNA-based phylogenic analysis (Table 1). Besides, reported fecal pollution indicators such as *Klebsiella oxytoca, Pantoea agglomerans*, and *Enterobacter aerogenes* were also isolated from the schmutzdecke [14].

**Table 1:** Number of isolates, observed genus, observed phylum, and %strain of opportunistic pathogen in two different sources: Hyung-San River and Schmutzdecke developed by Hyung-San River.

Source	Labeling	Number of Isolates	Observed genus	Observed Phylum	% Strain of Opportunistic Pathogens
Hyung-San River	H	17	*Novosphingobium,* *Catellibacterium,* *Aeromonas,* *Leclercia,* *Raoultella,* *Microbacterium,*	*Proteobacteria,* *Actinobacteria*	29%
Schmutzdecke (biofilm) of BSF	HB	20	*Novosphingobium,* *Sphingomonas,* *Bradyrhizobium,* *Klebsiella,* *Enterobacter,* *Aeromonas,* *Pantoea,* *Cloacibacterium,* *Streptomyces,* *Arthrobacter* *Microbacterium,* *Brevibacillus.*	*Proteobacteria* *Actinobacteria,* *Fermicutes, Bacteroidetes*	55%

### 3.2. Phylogenic Analysis of Isolated Strains from Samples

**Figure 2:**
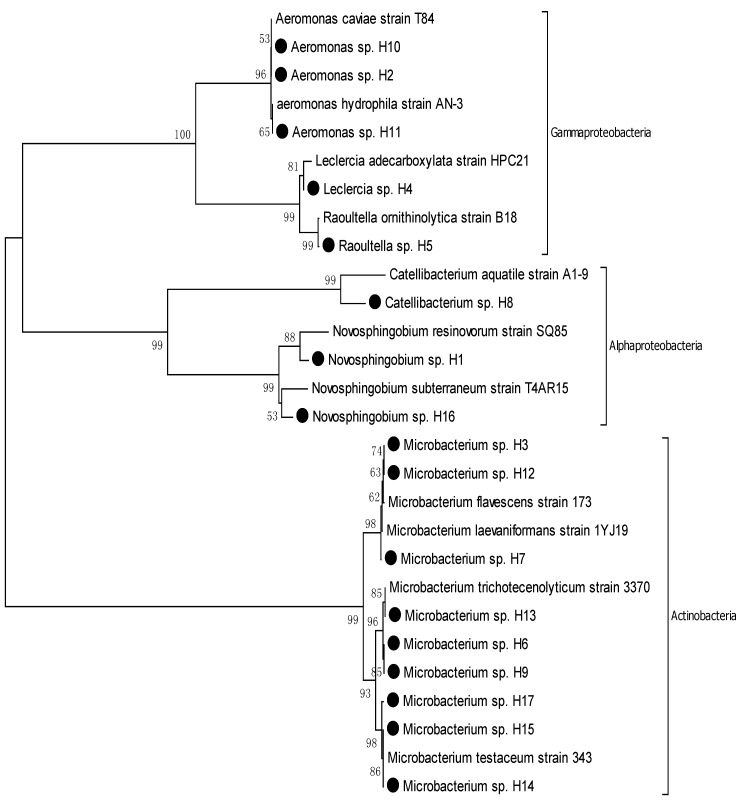
Phylogenic tree based on neighbor-joining analysis of 16S rRNA gene for Hyung-San River samples.

The nearest phylogenic neighbor of all 37 isolates from the Hyung-San River and schmutzdecke was determined by using the BLAST tool from NCBI based on the 16S rRNA gene sequence. The result of the BLAST analysis revealed that of all the strains, four strains belonged to the genus *Novosphingobium resinovorum*, one strain belonged to the genus *Catellibacterium,* two strains belonged to the genus *Sphingomonas,* two strains belonged to the genus *Bradyrhizobium,* four strains belonged to the genus *Bradyrhizobium,* one strain belonged to the genus *Leclercia,* one strain belonged to the genus *Raoultella,* one strain belonged to the genus *Klebsiella,* two strains belonged to the genus *Enterobacter*, two strains belonged to the genus *Pantoea,* three strains belonged to the genus *Cloacibacterium,* 10 strains belonged to the genus *Microbacterium,* two strains belonged to the genus *Streptomyces,* and one strain belonged to the genus *Brevibacillus* (Table 2 and Figure 2)*.*

The phylogenetic tree of the Hyung-San River species (Figure 2) shows the affiliation of 17 strains consisting of 11 different taxa. All the strains of the isolates from the Hyung-San River were divided into four Gram-positive groups and seven Gram-negative groups. All four Gram-positive groups, which include H3, H6, H7, H9, H12, H13, H14, H15, and H17, belonged to the phylum Actinobacteria. Of the seven Gram-negative groups, four groups, which include H2, H4, H5, H10, and H11, belonged to the phylum Gammaproteobacteria, and three groups, which include H1, H8, and H16, belonged to the phylum Alphaproteobacteria (Figure 2).

**Table 2:** Identification of 37 isolated strains from Hyung-San River and Schmutzdecke for their nearest phylogenic neighbors according to 16S rRNA gene sequence similarity%.

Sl No.	Strain No.	Nearest Phylogenic Neighbor	16S rRNA Gene Sequence Similarity %
		Gram-negative bacterial strains	
		*Proteobacteria*	
		*Alphaproteobacteria*	
1	H1	*Novosphingobium resinovorum* strain SQ85	98.1
2	H8	*Catellibacterium aquatile* strain A1-9	98.0
3	H16	*Novosphingobium subterraneum* strain T4AR15	98.2
4	HB12	*Novosphingobium sp*. HU1-AH51	98.8
5	HB13	*Sphingomonas sp*. M16	99.7
6	HB15	*Sphingomonas sp*. M16	99.6
7	HB17	*Novosphingobium aromaticivorans* DSM_12444	97.7
8	HB19	*Bradyrhizobium sp*. CCBAU 7128301	99.0
9	HB20	*Bradyrhizobium sp*. CCBAU 7128301	98.6
		*Gammaproteobacteria*	
10	H2	*Aeromonas hydrophila* strain AN-3	99.8
11	H4	*Leclercia adecarboxylata* strain HPC21	99.6
12	H5	*Raoultella ornithinolytica* strain B18	99.7
13	H10	*Aeromonas caviae* strain T84	99.7
14	H11	*Aeromonas hydrophila* strain AN-3	99.6
15	HB2	*Klebsiella oxytoca* strain LF-1	99.5
16	HB4	*Enterobacter aerogenes* strain DCH-2	99.5
17	HB5	*Aeromonas hydrophila* strain AN-3	99.7
18	HB6	*Pantoea agglomerans* strain 1BJN10	99.2
19	HB7	*Enterobacter cancerogenus* strain KNUC5008	98.9
20	HB14	*Pantoea agglomerans* strain 1BJN10	99.7
		*Bacteroidetes*	
		*Flavobacteria*	
21	HB8	*Cloacibacterium normanense* strain tu29	98.2
22	HB10	*Cloacibacterium rupense* strain R2A-16	97.9
23	HB18	*Cloacibacterium normanense* strain tu29	98.0
		Gram-positive bacterial strains	
		*Actinobacteria*	
		*Actinobacteridae*	
24	H3	*Microbacterium flavescens* strain 173	99.0
25	H6	*Microbacterium trichotecenolyticum* strain 3370	99.8
26	H7	*Microbacterium laevaniformans* strain 1YJ19	99.6
27	H9	*Microbacterium trichotecenolyticum* strain 3370	99.7
28	H12	*Microbacterium flavescens* strain 173	99.3
29	H13	*Microbacterium trichotecenolyticum* strain 3370	99.6
30	H14	*Microbacterium testaceum* strain 343	99.7
31	H15	*Microbacterium testaceum* strain 343	99.5
32	H17	*Microbacterium testaceum* strain 343	99.6
33	HB1	*Streptomyces sp*. MJM3179	99.8
34	HB3	*Arthrobacter oryzae* strain T42	99.2
35	HB9	*Microbacterium laevaniformans* strain 1YJ19	99.5
36	HB11	*Streptomyces sp*. MJM3179	99.9
		*Fermicutes*	
		*Bacilli*	
37	HB16	*Brevibacillus panacihumi* strain C17	99.4

**Figure 3:**
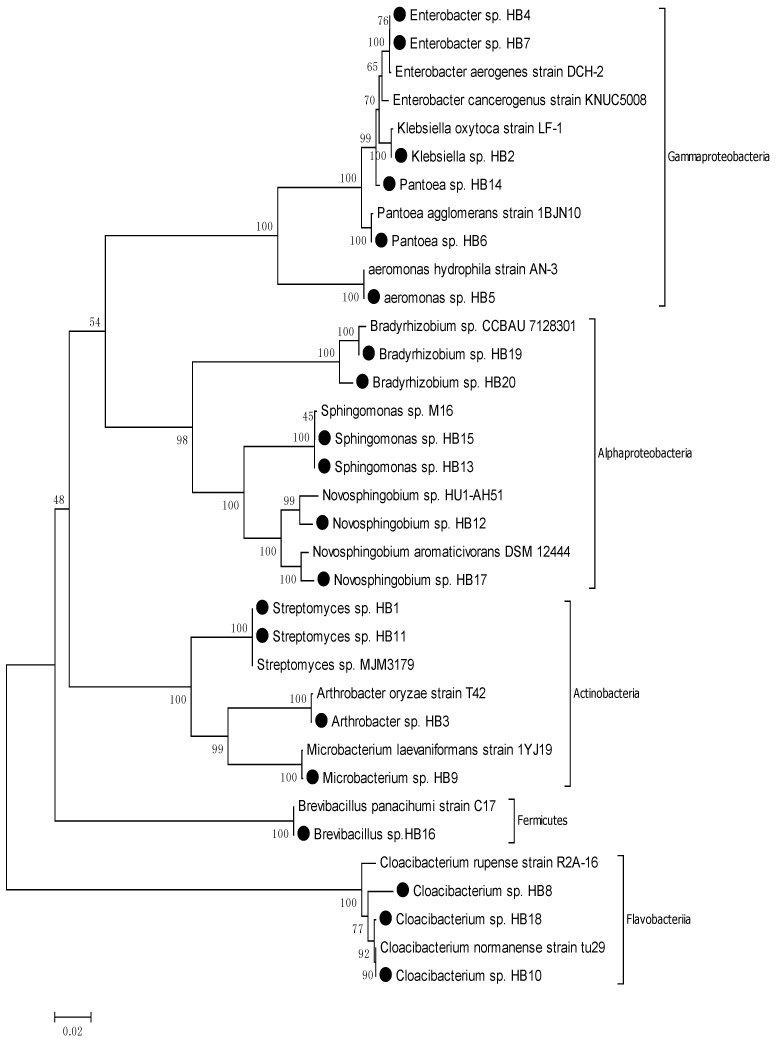
Phylogenic tree based on neighbor-joining analysis of 16S rRNA gene for schmutzdecke samples.

The phylogenic tree of the schmutzdecke (Figure 3) shows the affiliation of 20 strains consisting of 17 different taxa. All strains of the isolates from the schmutzdecke were divided into four Gram-positive groups and 13 Gram-negative groups. Of the four Gram-positive groups, three groups, which include HB1, HB3, HB9, and HB11, belonged to the phylum Actinobacteria, and one group, HB16, belonged to the phylum Firmicutes. Of the 13 Gram-negative groups, four groups, which include HB2, HB4, HB5, HB6, HB7, and HB14, belonged to the phylum Gammaproteobacteria; six groups, which include HB12, HB13, HB15, HB17, HB19, and HB20, belonged to the phylum Alphaproteobacteria; and three groups, which include HB8, HB10, and HB18, belonged to the phylum Bacteroidetes.

### 3.3. Identification of Opportunistic Pathogens and Their Associated Diseases

The nearest phylogenic neighbor of H2 and H11 was identified as *Aeromonas hydrophila* with 16S rRNA gene similarities of 99.8% and 99.6%, respectively. *Aeromonas hydrophila* is known to be a Gram-negative/facultative anaerobic bacteria and is considered to be a pathogen that induces mild diarrhea, life-threatening necrotizing fasciitis, septicemia, meningitis, cholera-like illness and hemolytic-uremic syndrome [15]. The nearest phylogenetic neighbor of H4 was identified as *Leclercia adecarboxylata* with 16S rRNA gene similarity of 99.6%. *Leclercia adecarboxylata* is Gram-negative/aerobic and is a pathogen that causes fever and leukocytosis [16]. The nearest phylogenic neighbor of H5 was identified as *Raoultella ornithinolytica* with 16S rRNA gene similarity of 99.7%. *Raoultella ornithinolytica* is known to be Gram-negative/aerobic and facultative anaerobic, and it induces enteric fever-like syndrome and bacteremia [17]. The nearest phylogenic neighbor of H10 was identified as *Aeromonas caviae* with 16S rRNA gene similarity of 99.7%. *Aeromonas caviae* is Gram-negative/facultative anaerobic and is known as a pathogen that causes gastrointestinal infectious diseases [18].

The nearest phylogenetic neighbor of HB1 and HB11 was identified as a *Streptomyces sp.* with 16S rRNA gene similarities of 99.8% and 99.9%, respectively. *Streptomyces* sp*.* are Gram-positive/aerobic and are known to induce general hypersensitivity [19]. The nearest phylogenetic neighbor of HB2 was identified as *Klebsiella oxytoca* with 16S rRNA gene similarity of 99.5%. *Klebsiella oxytoca* is Gram-negative/anaerobic and is considered to be a pathogen that causes septic arthritis [20]. The nearest phylogenic neighbor of HB4 was identified as *Enterobacter aerogenes* with 16S rRNA gene similarity of 99.5%. *Enterobacter aerogenes* is Gram-negative/facultative aerobic and is known to be a pathogen that induces a wide variety of infections [21]. The nearest phylogenic neighbor of HB5 was identified as *Aeromonas hydrophila* with 16S rRNA gene similarity of 99.7%. *Aeromonas hydrophila* is a known pathogen that causes symptoms previously mentioned. The nearest phylogenic neighbor of HB6 and HB14 was identified as *Pantoea agglomerans* with 16S rRNA gene similarity of 99.2% and 99.7%, respectively. *Pantoea agglomerans* is Gram-negative/aerobic and is a pathogen that causes soft tissue or bone/joint infections [22]. The nearest phylogenic neighbor of HB7 was identified as *Enterobacter cancerogenus* with 16S rRNA gene similarity of 98.9%. *Enterobacter cancerogenus* is Gram-negative/facultative anaerobic and causes wound and urinary tract infection, sepsis, and osteomyelitis [23]. The nearest phylogenic neighbor of HB17 was identified as *Novosphingobium aromaticivorans* with 16S rRNA gene similarity of 97.7%. *Novosphingobium aromaticivorans* is known to be Gram-negative/strictly aerobic and causes infection that induces autoimmune primary biliary cirrhosis [24]. Finally, the nearest phylogenic neighbor of HB13 and HB15 was identified as *Sphingomonas sp.* with 16S rRNA gene similarities of 99.7% and 99.6%, respectively. *Sphingomonas sp*. is known to be Gram-negative/aerobic and causes infectious disease [25] (Table 3).

**Table 3:** Identification of opportunistic pathogens and their associated human diseases.

Sl No.	Nearest Phylogenic Neighbor	Phylum	General Characteristics	Associated Human Disease	Reference
1	*Raoultella ornithinolytica* B6	Proteobacteria	Gram-negative, aerobic/facultative anaerobic	Enteric fever-like syndrome and bacteremia	Victoria Pulian Morais *et al.*, 2009 [15]
2	*Aeromonas caviae*, strain NCIMB 13016	Proteobacteria	Gram-negative, facultative anaerobic	Gastrointestinal infectious disease	Meiyanti *et al.*, 2010 [16]
3	*Klebsiella oxytoca* strain LF-1	Proteobacteria	Gram-negative, anaerobic	Septic arthritis	Mendard A *et al.*, 2010 [18]
4	*Enterobacter aerogenes* strain DCH-2	Proteobacteria	Gram-negative, facultative aerobic	All kinds of infections	Irene G *et al.*, 2007 [19]
5	*Pantoea agglomerans* strain 1BJN10	Proteobacteria	Gram-negative, aerobic	soft tissue or bone/joint infections	Andrea T *et al.*, 2007 [20]
6	*Enterobacter cancerogenus* strain KNUC5008	Proteobacteria	Gram-negative, facultative anaerobic	Wound and urinary tract infection, sepsis, and osteomyelitis	I. Stock *et al.*, 2002 [21]
7	*Novosphingobium aromaticivorans* DSM 12444	Proteobacteria	Gram-negative, strictly aerobic	Autoimmune primary biliary cirrhosis induced by infection	Mohammed JP *et al.*, 2011 [22]
8	*Aeromonas hydrophila* strain RB5-M1	Proteobacteria	Gram-negative, facultative anaerobic	Mild diarrhea, life-threatening necrotizing fasciitis, septicemia, meningitis, cholera-like illness, and hemolytic-uremic syndrome	Grim CJ *et al.*, 2013 [13]
9	*Leclercia adecarboxylata* strain HPC21	Proteobacteria	Gram-negative, aerobic	Fever and leukocytosis	Zelalem Temesgen *et al.*, 1997 [14]
10	*Streptomyces sp*. MJM3179	Actinobacteria	Gram-positive, aerobic	Hypersensitivity	Monk *et al.*, 2007 [17]
11	*Sphingomonas sp.* M16	Proteobacteria	Gram-negative, aerobic	Infectious disease	David C White *et al.*, 1996 [23]

## 4. Discussion

Our results indicate that schmutzdecke developed using water sourced from the Hyung-San River contains diverse and relatively high portions of opportunistic pathogens (55% of all HB isolates were identified as opportunistic pathogens). Moreover, the results demonstrate a tendency toward an increased proportion of opportunistic pathogens in the schmutzdecke compared to its water source, suggesting that pathogens in the water source are trapped and attached to the sticky layer of the schmutzdecke and accumulated on the surface of the sand while water flows through the filter [9] (% strain of the opportunistic pathogen increases from 29% in water sources to 55% in schmutzdecke). Our result is supported by the World Health Organization (WHO) report, which indicates that the microorganisms contained in water source attach to the surface of fine sand (Figure 1) and gradually accumulate to become part of the schmutzdecke [9]. However, in contrast with the WHO report, our study focused on pathogens more than on ordinary microorganisms. Concerning the underlying mechanism of schmutzdecke formation, as a whole, our study implies that the schmutzdecke developed by any water source containing pathogens could also contain opportunistic pathogens, as observed in our results; in other words, schmutzdecke acts as a trap for pathogens in the water source.

Moreover, diversity in the genus, phylum, and strains of opportunistic pathogens was increased in the schmutzdecke compared to the water sources. In the water source, six different genera were observed, while 12 different genera were observed in the schmutzdecke. *Novosphingobium, Aeromonas*, and *Microbacterium* were commonly found in both sample H and sample HB; however, *Sphingomonas*, *Bradyrhizobium*, *Klebsiella, Enterobacter*, *Pantoea*, *Cloacibacterium*, *Streptomyces*, *Arthrobacter*, and *Brevibacillus* were newly observed in schmutzdecke-originated samples (HB). Moreover, only two phyla (Proteobacteria and Actinobacteria) were observed in the water source, while four phyla (Proteobacteria*,* Actinobacteria*,* Firmicutes, and Bacteroidetes) were observed in the schmutzdecke. In the water source, four opportunistic pathogen strains (*Raoultella ornithinolytica*, *Aeromonas caviae*, *Aeromonas hydrophila*, and *Leclercia adecarboxylata*) were observed, whereas seven opportunistic pathogen strains (*Klebsiella oxytoca*, *Enterobacter aerogenes*, *Pantoea agglomerans*, *Enterobacter cancerogenus*, *Novosphingobium aromaticivorans*, *Aeromonas hydrophila*, and *Streptomyces sp*.) were observed in the schmutzdecke, indicating increased microbiological diversity in the schmutzdecke compared to the water source.

*Aeromonas hydrophila*, an opportunistic pathogen, was isolated from both schmutzdecke and raw water; on the other hand, there were strains that were isolated from either. Identified microorganisms in the schmutzdecke and raw water can differ because microorganisms from other sources besides raw water can also participate in the schmutzdecke. Microorganisms from other sources such as sand participate in the schmutzdecke, possibly inducing competition and microbial composition changes in the schmutzdecke.

Gomez-Villalba *et al.* applied a culture-independent method (temperature-gradient gel electrophoresis—TGGE) to analyze biofilm in a pilot-scale submerged biofilter and found that most of the species belonged to the Proteobacteria phylum [26]. This is consistent with our result that most of the species found in the schmutzdecke (biofilm) were Proteobacteria, perhaps indicating that Proteobacteria survive well in biofilm and water-associated conditions. Furthermore, Feng *et al.* reported that they detected the opportunistic pathogen *Sphingomonas* in the microbial community in the sand body portion of biosand filters and in the granular activated carbon-sand dual media filter portion by using 16S rRNA gene clone library analysis [27,28]. Although the work of Feng *et al.* was more focused on simple microbial communities at 0.2 m depth in the sand body of filters and our study mainly focused on the schmutzdecke (biofilm) in the upper portion of the fine sand, the work of Feng *et al.* is consistent with our results relating to *Sphingomonas* detection.

Our results demonstrate that the schmutzdecke developed with water sourced from the Hyung-San River contains diverse fecal pollution indicators, such as *Klebsiella oxytoca*, *Pantoea agglomerans*, and *Enterobacter aerogenes*, which are reported to cause various infections threatening public health. Because many of the water sources in developing countries or rural areas are more or less contaminated with excreta or endemic pathogens [1], it can be predicted that BSFs applied in those areas will develop schmutzdecke containing opportunistic pathogens. Opportunistic pathogens and associated diseases will vary depending on the water sources, but it is clear that careless treatment of schmutzdecke containing opportunistic pathogens will be a potential hazard to public health.

The schmutzdecke plays a key role in the biosand filter due to its biological purifying function. Since few pathogens were detected in the treated water according to the previous studies, people implicitly assume that neither will there be barely any pathogens in the schmutzdecke layer. It is more like common sense to assume no pathogen survives in the schmutzdecke due to its purifying effects and this can eventually lead to the careless treatment of the schmutzdecke waste. However, looking from a different angle, our study tried to estimate whether there really is no pathogenic microorganisms in the schmutzdecke and we found that there are diverse opportunistic pathogens in the schmutzdecke. Nonetheless, our study is still limited to explain what the influence of purifying mechanisms was on the pathogenic microorganisms that were identified. Therefore, the further study dealing with the underlying mechanism of the phenomenon is expected to be followed for better understanding of the schmutzdecke.

Because schmutzdecke clogs and prevents water flow through the filter as it develops, it should be cleaned or discarded on a regular basis to maintain the BSF [10]. Although schmutzdecke contains diverse opportunistic pathogens that have potential for infection/disease outbreak, there are no solid guidelines for the after-treatment of schmutzdecke. Due to their characterization as appropriate technology, BSFs are mainly managed by rural or native people after installation. If the natives do not receive any cautionary or guiding information about the after-treatment of schmutzdecke, it is probable that they will dump or pour schmutzdecke deposits into the water source with little awareness when recharging BSFs. Disposal of the schmutzdecke deposit in the water source or anywhere that can flow to the water source should be restricted. This is because schmutzdecke (biofilm) is an accumulated and condensed form of microorganisms, including opportunistic pathogens; additionally, biofilm is a protected form that survives better and longer in aqueous conditions [29,30]. Consequently, the negative impacts of pathogens are potentially amplified and prolonged, which, in extreme cases, could result in epidemic outbreaks. Even a small quantity of pathogens in a water source that previously caused no conspicuous harm to people could turn into a significant hazard as the microbes form schmutzdecke (biofilm) because biofilm has a resilient nature that leads to persistent infections in humans [29].

Our main purpose of the study is to spot the microbial status of schmutzdecke at the point when the schmutzdecke waste should be treated, and thus we selected single sampling event point when the schmutzdecke in our experimental filter is saturated. In terms of the schmutzdecke waste, the periodic point when the schmutzdecke waste should be discarded varies depending on the turbidity and the flow rate of water. However, generally, replacing of the schmutzdecke is required when it is saturated, and thus the flow rate drops to a level that is inadequate for the household use. Even though we tried to spot microbial status of the schmutzdecke when it is treated, our study is still limited to single sampling event. Therefore, the further study dealing with samples from diverse periodic points should be followed to trace microbial change of the schmutzdecke in the long term performance of the BSF.

As mentioned in the introduction, 12,346 institutions (this value includes only the official count) are supplying BSFs to more than 37 developing countries [5]. Although BSFs are widely applied in the field, there has been no study of the potential risks of schmutzdecke waste so far. Although our study does not include a variety of water sources, it still has significance in suggesting the potential risk of schmutzdecke and the necessity of creating guidelines or regulations for the after-treatment of schmutzdecke deposits. A cautious approach and careful attention are needed in treating schmutzdecke deposits. The results of the present study should be used as scientific background to revise and add guidelines for the after-treatment of schmutzdecke to BSF manuals. Once the manuals are revised and proper hygiene education is provided to local habitants by the BSF-supplying institutions, potential infections or epidemics caused by the careless treatment of schmutzdecke can be expected to be prevented.

## 5. Conclusions

We have evaluated the potential risk associated with schmutzdecke in biosand filters and its potential clinical impacts on human health in relation to diseases and infections. Opportunistic pathogens were identified by 16S rRNA-based phylogenetic analysis, and the results suggest that schmutzdecke, indeed, contains diverse opportunistic pathogens that are reported to be capable of causing infections in human. Rather than covering a variety of water sources, our study dealt the schmutzdecke developed using a specific water source to evaluate the potential risk of schmutzdecke deposits and the potential clinical impact in detail. However, further study of schmutzdecke developed using various water sources should be conducted to confirm the potential danger suggested by our study. Furthermore, although we referred to previously reported information to suggest the pathogenic effects of the isolates, this report does not necessarily confirm an actual pathogenic effect. Thus, readers should interpret our results as suggestive of the “potential” risk of schmutzdecke and its waste.
